# Breast Carcinoma: From Initial Tumor Cell Detachment to Settlement at Secondary Sites

**DOI:** 10.1155/2017/8534371

**Published:** 2017-07-12

**Authors:** Catharina Melzer, Juliane von der Ohe, Ralf Hass

**Affiliations:** Biochemistry and Tumor Biology Lab, Department of Obstetrics and Gynecology, Hannover Medical School, Hannover, Germany

## Abstract

Metastasis represents a multistep cascade of cancer cell alterations accompanied by structural and functional changes within the tumor microenvironment which may involve the induction of a retrodifferentiation program. Major steps in metastatic developments include (A) cell detachment from the primary tumor site involving epithelial-mesenchymal transition (EMT), (B) migration and invasion into surrounding tissue, (C) transendothelial intravasation into the vasculature of blood and/or lymphatic vessels as circulating tumor cells (CTCs), (D) dissemination to distant organs, and (E) extravasation of CTCs to secondary sites as disseminated tumor cells (DTCs). This article highlights some aspects of the metastatic cascade with a focus on breast cancer cells. Metastatic steps critically depend on the capability of cancer cells to adapt to distant tissues and the corresponding new microenvironment. As a consequence, increasing plasticity and developmental changes paralleled by acquisition of new cancer cell functionalities challenge a successful therapeutic approach.

## 1. Introduction

Breast cancer accounts for the most common type of cancer in women. The leading cause of cancer death results from metastasis and not from the primary tumor itself [1, 2].

**Figure 1:**
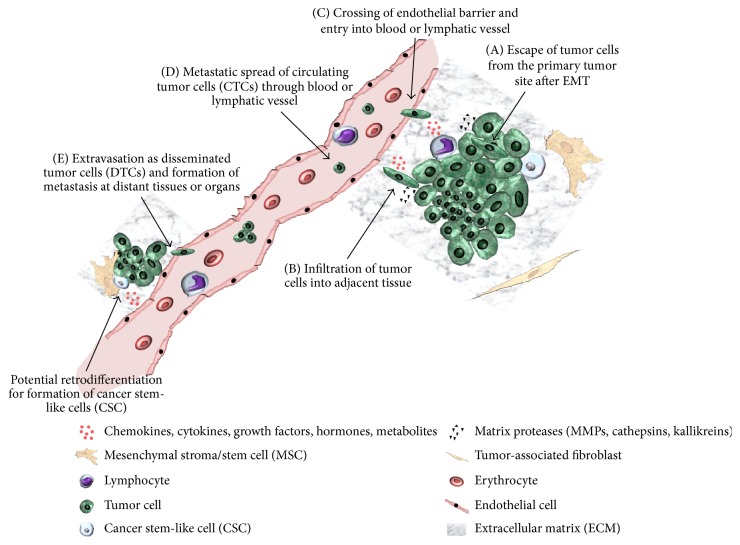
*Schematic view of metastatic process*. Metastatic cascade starting from primary tumor site to distant sites via (A) escape of tumor cells from the primary tumor site, (B) infiltration of tumor cells into adjacent tissue, (C) crossing of endothelial barrier and entry into blood or lymphatic vessel, (D) metastatic spread of circulating tumor cells (CTCs) through blood or lymphatic vessel, and (E) extravasation as disseminated tumor cells (DTCs) and formation of metastasis at distant site (modified according to [9]).

Breast cancer metastasis is characterized by a multistep cascade. This metastatic process can be subdivided into 5 steps which are discussed in principle and involve the following: (A) tumor cells including breast cancer stem-like cells are liberated from the primary tumor tissue potentially undergoing epithelial-mesenchymal transition (EMT), (B) tumor cells migrate and infiltrate neighboring tissue, (C) tumor cells cross endothelial barrier and enter blood and lymphatic vessels as circulating tumor cells (CTCs), (D) tumor cells attach at secondary sites after circulation to escape blood and lymphatic vessels as disseminated tumor cells (DTCs), and (E) tumor cells migrate to distant tissue and form metastases [3–5] (see Figure 1). Especially for the first step of the metastatic cascade, the tumor microenvironment (TME) has a tremendous impact whereby direct and indirect interactions contribute to further development and heterogeneity of the breast tumor, including progression and initiation of metastasis. The TME harbors several cell populations such as a variety of different immune cells, pericytes in perivascular niches, mesenchymal stroma/stem cells (MSC), tumor-associated fibroblasts, adipocytic cells and endothelial precursors, and mature cells. Moreover, soluble factors like cytokines, chemokines, growth factors, hormones, metabolites, and components of the extracellular matrix (ECM) additionally contribute to tumor maturation and diversification. Of interest, particular interaction of MSC with breast cancer cells favors the establishment of a putative carcinoma stem cell niche for generation of cancer stem cell-like cells (CSCs) or tumor-initiating cells (TICs) [6–11]. Although various studies consider CSCs as TICs [12, 13], other work discriminated this interchangeability by stem cell markers, for example, CD133-expressing CSCs in the colon or CD24^low^/CD44^high^ and ALDH1^high^ expression by breast cancer CSCs representing different functional characteristics [as reviewed in [14]]. Moreover, tumor growth and gene expression profiles in CSCs of metastases are significantly altered as compared to a TIC in the primary tumor which could be more appropriately described as originating tumor cell [as reviewed in [15, 16]]. Cellular processes for a successful development of metastases are performed by several strategies and diversifications which can vary within different tumor entities. Accordingly, the present work mainly focuses on formation of breast cancer metastases.

*(A) Tumor Cells Escape from the Primary Tumor Site*. The beginning of metastasis is represented by detachment of individual cells from the primary tumor site [17, 18]. Since detached epithelial and endothelial cells undergo anoikis due to incorrect ECM/cell attachment, detached cancer cells display a certain resistance to anoikis [19]. In order to escape apoptosis tumor cells alter phenotype and functionality including loss of cell polarity and changes in cell-to-cell and cell-matrix adhesion and an increase in migratory potential. These functional and structural alterations are achieved via induction of EMT in the cancer cells [20, 21]. However, there is evidence that EMT is not essential for metastasis. In vivo mice studies revealed that lung metastases exhibit non-EMT cancer cells, that inhibition of EMT influences lung metastasis formation, but that additionally EMT cells show chemoresistance and are the cause for recurrence [22]. Similar results have been reported with pancreatic ductal adenocarcinoma whereby EMT suppression did not affect metastasis but led to induction of chemoresistance [23].

EMT can be initiated by autocrine and paracrine signals involving TGF-*β* and Wnt or by activation of receptor tyrosine kinases via binding and trans-signaling of growth factors such as epidermal growth factor or fibroblast growth factor [24]. In general, EMT induction leads to activation of EMT-associated transcription factors including Twist1, Slug, Zeb1/2, and Snail1/2 which promote downregulation of, for example, E-cadherin. Consequently, tumor cells lose cell-to-cell adhesion and reduce cell-cell junctions [25]. Moreover, mesenchymal markers like fibronectin, vimentin, and N-cadherin become activated which leads to a more mesenchymal-like phenotype with enhanced migration and increased cell-to-stroma interactions [26–28].

The acquisition of mesenchymal marker expression has also been reported in several studies addressing interactions between mesenchymal stroma/stem cells (MSC) and cancer cells including breast and ovarian cancer [29]. Indeed, MSC represent a heterogeneous cell population with multiple subpopulations displaying stem cell-like properties [30, 31]. Whereas MSC reside in a variety of different tissues within the organism including bone-marrow, adipose tissues, peripheral blood, dental pulp, and perivascular niches of several other tissues, predominant properties include differentiation capacity along phenotypes of the mesenchymal lineage and potential cross-germline maturation. Previous studies suggested that MSC from birth-associated tissues such as umbilical cord exhibit superior properties including a higher expansion rate and engraftment capacity as compared to MSC derived from adult tissue sources [as reviewed in [32]]. Moreover, MSC exhibit immune-modulatory functions at sites of tissue damage and injury. Thereby, local tissue-associated MSC contribute to wound healing, damage repair, and tissue regeneration and homeostasis by secretion of anti-inflammatory cytokines, chemokines, metabolites, and a large variety of growth factors which in parallel can stimulate endothelial cells for vessel formation and subsequent neovascularization. These multiple functionalities of MSC and their crosstalk with various adjacent cell populations which depends on the state of activation and the age of MSC also apply to cellular interactions between MSC and neighboring tumor cells, considering invasive cancer growth as a permanent wound [32–34]. Therefore, MSC also play an important role during tumor cell interaction. For example, breast cancer cells acquire CD90 during coculture with umbilical cord-derived MSC involving gap junctional intercellular communication and Notch signaling which represents one of the characteristic mesenchymal markers of MSC [35]. Moreover, interactions between MSC and cancer cells proceed bidirectionally since vice versa, acquisition of some epithelial-like markers including epithelial cell adhesion molecule (EpCAM) can be detected in MSC following coculture with ovarian cancer cells [36]. Furthermore, MSC acquire increased expression of ECM proteins such as fibronectin and laminin during certain chemotherapeutic treatment and thereby promote tumor cell protection and a distinct chemoresistance [37].

In addition to the downregulation of epithelial traits and upregulation of certain mesenchymal markers in cancer cells, release of ECM-degrading enzymes including matrix metalloproteinases (MMPs) facilitates liberalization from the originating tumor tissue, migration, and subsequent invasion into tumor-neighboring tissue such as epithelial cell layers and eventually blood or lymphatic vessels (Figure 1).

Together, interactions between MSC and tumor cells can facilitate a mesenchymal-like transition of cancer cells to support EMT and metastatic potential.

*(B) Tumor Cells Migrate and Infiltrate Neighboring Tissue*. Tumor cells can migrate either individually and/or collectively through adjacent connective tissue. Single cell movement can be performed slowly via a mesenchymal type of migration. Alternatively, tumor cells display a faster movement via so-called amoeboid cell invasion. By contrast, leader cells of collectively migrating cancer cells exhibit a mesenchymal migration phenotype whereby inner cells of the collectively migrating unit retain their epithelial phenotype [3, 38].

Mesenchymal movement encompasses a five-step migration cycle characterized by pseudopod protrusion at the leading edge, formation of focal contacts, focalized proteolysis, actomyosin contraction, and detachment of the trailing edge [as reviewed in [39]]. This type of invasion mainly arises in cells which undergo EMT from epithelial cancers or connective tumor tissue. In contrast to amoeboid migration, mesenchymal invasion is protease dependent since it involves a plethora of soluble, secreted, and surface proteases including matrix metalloproteinases (MMPs), kallikreins, serine proteases, and cathepsins to enzymatically remodel and cleave ECM components such as collagen, fibronectin, and laminin to enable movement through the ECM [39, 40]. Cathepsins are found both extracellularly and intracellularly in lysosomes and are represented by different amino acids at active sites such as serine (cathepsin G), aspartic acid (cathepsin D), and cysteine (cathepsins B, F, H, K, and L) [41]. Whereas secreted cathepsins cleave extracellular ECM components, intracellularly located cathepsins degrade endocytosed ECM proteins like collagen in lysosomes [42–46]. For instance, inhibition of cathepsin B reduced ECM degradation and decreased inflammatory breast cancer invasion in vitro [47]. Besides proteolytic enzymes like MMPs and cathepsins, kallikreins represent a further protease family of ECM remodeling proteins. Evidences suggest that kallikreins are involved in tumor progression and distinct kallikreins are applied as tumor marker in cancer diagnosis, for example, kallikrein hK3 also known as prostate-specific antigen [48, 49]. Moreover, the human kallikreins hK2 and hK4 can induce activation of the urokinase plasminogen activator system resulting in ECM degradation and/or activation of tumor cell spreading [48, 50].

Particularly MMPs play an essential role in cancer cell invasion by reorganization of the ECM which consists of basement membranes and interstitial matrix. For example, MMP2 and MMP9 degrade collagen type IV, the major component of basement membranes representing thin structures at the basolateral site of epithelium and endothelium [51, 52]. Moreover, MMP7 was suggested in juvenile human mammary epithelial cells (HMEC) to form a ternary complex together with the growth factor precursor form pro-HB-EGF and anchorage to the hyaluronan receptor CD44. Subsequent cleavage releases the soluble sHB-EGF which binds to the dimerized form of the ErbB4 surface receptor to relay intracellular signaling via the cleaved 4ICD intracellular receptor domain and phosphorylation of Erk1/2, thereby activating Fra-1 for nuclear translocation and repression of tropoelastin production. Vice versa, senescent HMEC express reduced MMP7 protein whereby formation of the ternary complex with HB-EGF and CD44 is disrupted. The resulting failure to activate Fra-1 via the missing ICD induces tropoelastin formation, subsequent cleavage by lysyl oxidases, and extracellular formation of elastin fibers. Consequently, signaling in senescent HMEC with altered MMP7 levels has been attributed to increased fibrosis as a prerequisite for breast cancer development [53–55].

Besides MMP2, MMP7, and MMP9, transmembrane collagenase MT1-MMP/MMP14, which is particularly associated with cell protrusions such as invadopodia, represents a key enzyme in remodeling ECM by driving invasion of tumor cells via degradation of collagen type I and thereby facilitating dissemination of cancer cells [40, 56–59]. Moreover, this MMP was demonstrated to protect invading breast carcinoma cells from collagen type I induced apoptosis [60].

In contrast to mesenchymal cell invasion, hallmarks of amoeboid cancer cell movements are protease independency with total loss of cell polarity, lower adhesiveness due to missing focal contacts, and reduced capability to remodel the ECM. Furthermore, amoeboid migrating tumor cells grow in suspension and exhibit roundish cell morphology [39, 61, 62]. These characteristics allow amoeboid-like invading tumor cells to move much faster through adjacent tissue compared to mesenchymal movement [63, 64].

Both migration types can be performed by single invading tumor cells. Additionally, carcinoma cells exhibit the ability to migrate collectively during metastasis. Collective invasion is characterized by maintenance of cell-to-cell adhesion and front-rear polarity within the migrating cell unit by multicellular coordination and generation of traction force for forward migration and by remodeling of ECM. The plasticity of collectively migrating tumor cells ranges from coordinated sheets, strands, and tubes to clusters [65–68]. Nevertheless, tip cells or leader cells, respectively, feature mesenchymal morphology, whereas subsequent cells are structured by epithelial cell-to-cell contacts. One major advantage of collective cell movement is the protection of inner cells, for example, from immune cell assaults [as reviewed in [39, 62]].

However, all the different tumor cell movements are not mutually exclusive. Switch from mesenchymal-to-amoeboid motility and vice versa can occur under certain conditions and is designated as mesenchymal-to-amoeboid transition (MAT) or amoeboid-to-mesenchymal transition (AMT) [69, 70]. In addition, collective to amoeboid transition (CAT) has been demonstrated in melanoma [65]. Of interest, breast cancer cells have been reported to use both migration types, single and collective cell movement, during metastatic development [71].

Even though cancer cells which are detached from primary tumor site experience a completely different microenvironment while invading through adjacent tissue, they undergo structural and functional changes that allow infiltration and forward movement to attain access to blood and lymphatic vessels.

Chemokines and further soluble factors play an important role in guiding migration of cancer cells towards vasculature and contributing to the metastatic spread [72]. Particularly, chemokine receptor CCR7 has been reported to mediate migration to lymph nodes in gastric and colorectal carcinoma [73, 74]. Breast cancer cells expressing chemokine receptors CXCR4 and CCR7 were stimulated with appropriate chemokine receptor ligands CCL12 and CCL21 leading to pseudopodia formation and higher invasiveness [75]. Additionally, siRNA knock-down of CXCR4 in breast cancer cells or neutralizing antibody against CXCR4 reduced tumor growth and inhibited metastasis [75, 76]. The CXCR4/CXCL12 axis is used by tumor cells which secrete platelet-derived growth factor (PDGF) that in turn activates endothelial cells to secrete CXCL12 leading to a chemotaxis gradient [77]. This gradient may attract CXCR4 positive tumor cells to the endothelium approaching the vasculature. Moreover, breast cancer-mediated production of the chemokine CCL5 (RANTES) by neighboring tumor-associated mesenchymal stroma/stem cells can act in a paracrine manner on the cancer cells to promote their motility, invasion, and metastasis [78].

*(C) Tumor Cells Cross Endothelial Barrier and Enter Blood and/or Lymphatic Vessels*. Cancer cells detached from the primary tumor site can utilize both the blood and lymphatic system to spread to secondary sites for metastasis [79]. Prerequisites for this development are neovascularization of the tumor site or lymphangiogenesis [80, 81] to provide appropriate vessel structures within the tumor microenvironment.

To enter the blood or lymphatic system and of course to leave this system again, cancer cells need to cross the endothelial barrier. Disruption of this barrier is indispensable and involves various mechanisms which cancer cells use for intra- and extravasation [82–84].

One mechanism suggests that endothelial barrier leakage occurs via tumor cell-secreted factors and receptor-ligand interactions. Metastatic melanoma cells induced endothelial cell gap formation via Src activation in endothelial cells, a nonreceptor tyrosine kinase. Further activation of Src was mediated by tumor cell-derived IL8 and VLA-4/VCAM-1 interactions leading to phosphorylation of VE-cadherin, a major component of endothelial junctions and finally to disruption of the endothelial barrier [83]. Other in vivo studies suggested a potential role for VEGF in promoting vascular permeability upstream of Src and VE-cadherin by applying VEGF and Src inhibitors that suppress tumor cell extravasation [84]. Moreover, epidermal growth factor receptor (EGFR) has been attributed to a sustaining role of tumor cell intravasation and dissemination [85].

There is still ongoing debate about the entry mechanism of tumor cells into vessels whether it occurs actively or passively. As outlined previously, motile cancer cells can enter the blood and lymphatic system actively while mobile cells are moved by external forces, for instance, mechanical tension through the endothelium into blood and lymphatic vessels [17, 86, 87]. Motile tumor cells invade either individually or collectively. For both ways, tumor cells have to undergo significant changes involving EMT to acquire higher migratory potential concomitant with altered cell morphology and a phenotype with certain stem cell characteristics similar to CSCs or TICs [17, 88–94].

Tumor cells that have escaped the primary tumor site and entered blood or lymphatic system for metastatic spread are also defined as circulating tumor cells (CTCs) [95].

*(D) Tumor Cells Disseminate to Secondary Sites as Circulating Tumor Cells (CTCs)*. CTCs have to overcome several obstacles that implicate blood and lymphatic vessels. Indeed, the vascular environment is completely different compared to the TME of the primary tumor site and is responsible for high mortality rates of CTCs [96]. Clinical studies about CTC occurrence in breast cancer patients revealed an averaged number of around 80 CTCs per mL blood [97] equivalent to nearly half a million of CTCs in the whole circulation. This actually demonstrates a certain inefficiency and limitation of the metastatic process whereby reasons are partially due to the vascular environment. However, prognostic values for appearance of metastatic breast CTCs/mL by diagnostic approaches of CTC enrichment technologies using liquid biopsy could be much lower and may depend on the genomic heterogeneity of CTCs and individual patient conditions [98, 99].

First of all, fluid shear stress, the mechanical force of the blood flow, and frequent collisions with blood and immune cells represent one major hindrance for CTCs to survive and to reach distant organs [17, 100]. Nonetheless, it was shown that CTCs which underwent EMT are more resistant against these kinds of insults than tumor cells with an epithelial phenotype [96]. Collisions of CTCs with blood cells mainly result from high numbers of leukocytes, erythrocytes, and platelets affecting CTC viability [96, 101]. Moreover, CTC survival requires rescue or self-defense against immune cell assaults, particularly those from natural killer (NK) cells [102]. CTCs can evade the effective antitumoral activity of NK cells via induction of platelets to aggregate, representing a potential mechanism of tumor cells for platelet activation (tumor cell-induced platelet aggregation, TCIPA) [103, 104]. TCIPA proceeds directly via interaction of platelets with CTCs or indirectly via soluble molecules like cysteine proteases or ADP [105, 106]. Moreover, MMPs play an essential role in TCIPA and cancer cells may use this possibility to circumvent NK cell activity. For instance, in vitro studies showed that MCF7 breast cancer cells cotransfected with MMP14/*β*3-integrin caused platelet aggregation via introduced MT1-MMP/MMP14 and via activation of MMP2. Additionally, ADP contributed to TCIPA via stimulation of the corresponding platelet receptor P2Y_12_ [107, 108]. Thus, it is feasible that tumor cell-induced platelet aggregation promotes survival of cancer cells in vascular circulation since platelets may shield CTCs from immune cell assaults and from fluid shear stress and may facilitate extravasation at distant organs resulting in enhanced metastatic potential [109–111].

In addition to TCIPA to protect from antitumoral immune cell activity, tumor cells can selectively reduce tumor-suppressive TGF-*β*-signaling facilitating tumor progression through the suppression of the host immune system [112, 113]. For instance, in vivo studies revealed antitumor effects through T-cell specific inhibition of TGF-*β*-signaling [114].

A further obstacle for CTCs is the lack/deficiency of cell-matrix interactions to provide proliferation signals and cellular stability. Nevertheless, CTCs that have undergone EMT and acquired a mesenchymal-like phenotype do not require such interactions for cell survival [17, 88, 115].

All these hindrances of an altered microenvironment in the vascular system lead to an overall low survival rate of CTCs in the vasculature. For instance, it has been demonstrated that circulating breast cancer cells survive only a few hours in the circulation [116]. In most patients with advanced cancer (stage IV) the occurrence of CTCs is around 1 cell per billion normal blood cells [117]. Accordingly, this low number emphasizes the difficulties and challenges to detect CTCs in early stage cancer patients for timely diagnosis and therapy.

*(E) Tumor Cells (CTCs) Escape Blood and Lymphatic Vessels and Migrate as Disseminated Tumor Cells (DTCs) to Secondary Sites*. Extravasation starts with a reduction in blood flow velocity and a corresponding reduced CTC circulation in smaller capillaries in order to facilitate blood vessel wall attachment [118, 119]. There are two mechanisms for CTC extravasation, (1) physical occlusion and (2) cell adhesion. Whereas physical occlusion takes place in capillaries with a diameter smaller than CTCs, cell adhesion occurs in larger capillaries and requires direct binding to the endothelium [120, 121].

CTC adhesion to endothelial cells necessitates the expression of appropriate ligands and receptors on both CTCs and endothelial cells including cadherins, selectins, integrins, the hyaluronan receptor CD44, and immunoglobulin superfamily receptors [118, 122]. CD44 expressed on breast cancer cells serves as a major ligand for endothelial cell surface located E-selectin and mediates CTC adhesion to the endothelium [123]. Whereas endothelial selectin and cancer cell surface-expressed CD44 mediate CTC attachment, Rac1 and cell division control protein 42 (CDC42) promote transendothelial migration via extension of cancer cell protrusions facilitating the whole process of extravasation, for example, invadopodia which expand through the endothelial barrier [118, 124, 125]. Besides the interaction of breast cancer cell-derived integrin *α*v*β*3 with platelets, previous work demonstrated that integrin *α*v*β*3 also promotes CTC attachment to the endothelium in an activation-dependent manner [126, 127].

Following CTC attachment, metastasizing tumor cells cross endothelium by distinct mechanisms targeting endothelial junctions including VE-cadherin. Furthermore, cancer cells can incorporate into the endothelium displacing endothelial cells and disrupting the structure of the endothelium. Whereas neighbored endothelial cells maintain expression of VE-cadherin, endothelial cells in contact with cancer cells do not express VE-cadherin which may facilitate additional cancer cells to incorporate [128].

Breast cancer cells preferentially disseminate to lung, bone, liver, and brain [129, 130]. In particular, breast cancer cells with stem cell-like properties including CD44^high^/CD24^low^ subpopulations represent candidates for metastatic activities since this cell population can differentiate, escape immune surveillance, display apoptosis resistance, and sustain cell growth and self-renewal [90, 131–134]. Thus, different single cancer cell progenies acquire the capability to metastasize to distinct tissues/organs with features of an organ-specific homing whereby early dissemination of cancer cells favors metastatic capacity [2, 130, 135].

During EMT some tumor cells acquire characteristics and develop properties of CSCs [89, 90]. Nonetheless, the new microenvironment, which significantly differs between the different tissues/organs, is important for survival of extravasated CTCs and will be, most commonly, hostile for CTCs. Friendly microenvironments may represent metastatic niches which harbor stromal cell types including MSC and certain ECM proteins. Such a carcinoma stem cell niche may provide conditions via diverse tumor cell interactions or induction of a retrodifferentiation process to reprogram tumor cells for CSC formation and protection [9, 136]. Development of breast cancer stem-like cells are associated with expression of low levels of CD24, high levels of CD44, aldehyde dehydrogenase, and the IL8-binding chemokine receptor CXCR1 [137–140].

Newly formed CSCs can be kept in a dormant/quiescent state and therefore elevate the chance for CTC survival via cell-to-cell and cell-to-matrix interactions [10, 88, 141, 142]. Although CTCs that underwent EMT exhibit a higher migratory potential and invasiveness, their proliferative capacity and ability for cell-to-cell interactions remain limited [143]. Consequently, only a subset of CTCs will survive as disseminated tumor cells (DTCs) while the majority may die or reside as dormant cells [129, 144].

Mesenchymal-to-epithelial transition (MET), the reverse process of EMT at primary tumor sites, is deliberated to favor colonization of DTCs which can occupy distant organs as solitary cells, small preangiogenic metastases, or greater vascularized metastases [94, 129, 145]. Crucial signaling pathways to initiate MET involve protein kinase A (PKA) activation and following nuclear translocation. Subsequent PKA-mediated phosphorylation and thus activation of the histone demethylase PHF2 promote transactivation of epithelial cell-associated genes and protein products such as E-cadherin [146]. One hallmark of EMT is the downregulation of E-cadherin resulting in loss of cell adhesion [25]. Accordingly, reexpression of E-cadherin favors colonization of DTCs at distant organs or tissues. Recent studies revealed the expression of E-cadherin in metastases of E-cadherin-negative breast cancer xenografts induced by a secondary organ microenvironment [147]. Moreover, the reciprocal interplay of ZEB1/miR200 has been suggested as regulation of EMT and MET in cancer. Whereas transcription factor ZEB1 is known as potential inducer of EMT via repression of miR200 family members, miR200 has been reported to induce MET by repression of ZEB1 which subsequently leads to higher expression of E-cadherin [148–150].

In some breast cancer patients, metastases can arise after a long time, even after years or decades following first diagnosis. Since the majority of DTCs may reside as dormant cells in metastatic niches, local neovascularization together with a certain chemokine/metabolite/growth factor cocktail can stimulate reentry into the proliferative cell cycle and contributes to cancer relapse. Indeed, breast cancer cell models revealed that endothelial cells induce and maintain dormancy of DTCs in metastatic niches via thrombospondin-1, known as an inhibitor of angiogenesis and tumor growth [151, 152]. Moreover, time-lapse studies indicated that neovascular tips promote breast cancer growth and that these neovascular tips are rich in periostin and TGF-*β*1 suggesting an additional role for these two soluble factors in cancer relapse [151].

## 2. Concluding Remarks

The plasticity of tumor tissues and the continuous alterations/adaptations during liberation of tumor cells from the original tissue, transendothelial migration, and development of metastases include multiple changes in tumor cell phenotype and functionality. These variations challenge tumor cells to adapt to a new tissue environment with a certain threshold of chemokines, tissue-specific metabolites, and other soluble factors. Moreover, variations also include formation of new tumor cell populations, for example, by fusion or entosis with MSC during cellular interactions of adjacent cell types. All of these variations which are acquired in the course of metastasis contribute to the heterogeneity of the tumor and necessitate various tumor cell type-specific markers to provide potentially successful targets. Consequently, early interference with signaling pathways associated with tumor cell migration, spreading of CSCs, and formation of metastases represents a more promising therapeutic approach.

## Abbreviations

TME: Tumor microenvironment
EMT: Epithelial-to-mesenchymal transition
MET: Mesenchymal-to-epithelial transition
HMEC: Human mammary epithelial cells
MMP: Matrix metalloproteinase
MAT: Mesenchymal-to-amoeboid transition
AMT: Amoeboid-to-mesenchymal transition
CAT: Collective to amoeboid transition
MSC: Mesenchymal stroma/stem cells
CTCs: Circulating tumor cells
DTCs: Disseminated tumor cells
CSCs: Cancer stem cell-like cells
TICs: Tumor-initiating cells
NK cells: Natural killer cells
TCIPA: Tumor cell-induced platelet aggregation.

## References

[1] Jemal A., Bray F., Center M. M., Ferlay J., Ward E., Forman D. (2011). Global cancer statistics. *CA: A Cancer Journal for Clinicians*.

[2] Weigelt B., Peterse J. L., van't Veer L. J. (2005). Breast cancer metastasis: markers and models. *Nature Reviews Cancer*.

[3] Guan X. (2015). Cancer metastases: challenges and opportunities. *Acta Pharmaceutica Sinica B*.

[4] Kang Y., Pantel K. (2013). Tumor cell dissemination: emerging biological insights from animal models and cancer patients. *Cancer Cell*.

[5] Ma L., Teruya-Feldstein J., Weinberg R. A. (2007). Tumour invasion and metastasis initiated by microRNA-10b in breast cancer. *Nature*.

[6] Balkwill F. R., Capasso M., Hagemann T. (2012). The tumor microenvironment at a glance. *Journal of Cell Science*.

[7] Hass R., Otte A. (2012). Mesenchymal stem cells as all-round supporters in a normal and neoplastic microenvironment. *Cell Communication and Signaling*.

[8] Li H.-J., Reinhardt F., Herschman H. R., Weinberg R. A. (2012). Cancer-stimulated mesenchymal stem cells create a carcinoma stem cell niche via prostaglandin E_2_ signaling. *Cancer Discovery*.

[9] Melzer C., von der Ohe J., Lehnert H., Ungefroren H., Hass R. (2017). Cancer stem cell niche models and contribution by mesenchymal stroma/stem cells. *Molecular Cancer*.

[10] Melzer C., Yang Y., Hass R. (2016). Interaction of MSC with tumor cells. *Cell Communication and Signaling*.

[11] Ungefroren H., Sebens S., Seidl D., Lehnert H., Hass R. (2011). Interaction of tumor cells with the microenvironment. *Cell Communication and Signaling*.

[12] Qureshi-Baig K., Ullmann P., Haan S., Letellier E. (2017). Tumor-Initiating Cells: a criTICal review of isolation approaches and new challenges in targeting strategies. *Molecular Cancer*.

[13] Lichner Z., Saleh C., Subramaniam V., Seivwright A., Prud'homme G. J., Yousef G. M. (2015). miR-17 inhibition enhances the formation of kidney cancer spheres with stem cell/tumor initiating cell properties. *Oncotarget*.

[14] Hill R. P., Perris R. (2007). "Destemming" cancer stem cells. *Journal of the National Cancer Institute*.

[15] Chaffer C. L., Weinberg R. A. (2015). How does multistep tumorigenesis really proceed?. *Cancer Discovery*.

[16] Plaks V., Kong N., Werb Z. (2015). The cancer stem cell niche: how essential is the niche in regulating stemness of tumor cells?. *Cell Stem Cell*.

[17] Joosse S. A., Gorges T. M., Pantel K. (2015). Biology, detection, and clinical implications of circulating tumor cells. *EMBO Molecular Medicine*.

[18] Malin D., Strekalova E., Petrovic V. (2015). ERK-regulated *α*B-crystallin induction by matrix detachment inhibits anoikis and promotes lung metastasis in vivo. *Oncogene*.

[19] Douma S., Van Laar T., Zevenhoven J., Meuwissen R., Van Garderen E., Peeper D. S. (2004). Suppression of anoikis and induction of metastasis by the neurotrophic receptor TrkB. *Nature*.

[20] Liu S., Ye D., Guo W. (2015). G9a is essential for EMT-mediated metastasis and maintenance of cancer stem cell-like characters in head and neck squamous cell carcinoma. *Oncotarget*.

[21] Shang Z., Cai Q., Zhang M. (2015). A switch from CD44+ cell to EMT cell drives the metastasis of prostate cancer. *Oncotarget*.

[22] Fischer K. R., Durrans A., Lee S. (2015). Epithelial-to-mesenchymal transition is not required for lung metastasis but contributes to chemoresistance. *Nature*.

[23] Zheng X., Carstens J. L., Kim J. (2015). Epithelial-to-mesenchymal transition is dispensable for metastasis but induces chemoresistance in pancreatic cancer. *Nature*.

[24] Scheel C., Eaton E. N., Li S. H.-J. (2011). Paracrine and autocrine signals induce and maintain mesenchymal and stem cell states in the breast. *Cell*.

[25] Taube J. H., Herschkowitz J. I., Komurov K. (2010). Core epithelial-to-mesenchymal transition interactome gene-expression signature is associated with claudin-low and metaplastic breast cancer subtypes. *Proceedings of the National Academy of Sciences of the United States of America*.

[26] Angelucci C., Maulucci G., Lama G. (2012). Epithelial-stromal interactions in human breast cancer: effects on adhesion, plasma membrane fluidity and migration speed and directness. *PLoS ONE*.

[27] Wang M., Ren D., Guo W. (2016). N-cadherin promotes epithelial-mesenchymal transition and cancer stem cell-like traits via ErbB signaling in prostate cancer cells. *International Journal of Oncology*.

[28] Yan X., Yan L., Liu S., Shan Z., Tian Y., Jin Z. (2015). N-cadherin, a novel prognostic biomarker, drives malignant progression of colorectal cancer. *Molecular medicine reports*.

[29] Yang Y., Bucan V., Baehre H., Von Der Ohe J., Otte A., Hass R. (2015). Acquisition of new tumor cell properties by MSC-derived exosomes. *International Journal of Oncology*.

[30] Majore I., Moretti P., Hass R., Kasper C. (2009). Identification of subpopulations in mesenchymal stem cell-like cultures from human umbilical cord. *Cell Communication and Signaling*.

[31] Lavrentieva A., Majore I., Kasper C., Hass R. (2010). Effects of hypoxic culture conditions on umbilical cord-derived human mesenchymal stem cells. *Cell Communication and Signaling*.

[32] Hass R., Kasper C., Böhm S., Jacobs R. (2011). Different populations and sources of human mesenchymal stem cells (MSC): a comparison of adult and neonatal tissue-derived MSC. *Cell Communication and Signaling*.

[33] Hoffmann A., Floerkemeier T., Melzer C., Hass R. (2016). Comparison of *in vitro*-cultivation of human mesenchymal stroma/stem cells derived from bone marrow and umbilical cord. *Journal of Tissue Engineering and Regenerative Medicine*.

[34] Yang Y., Melzer C., Bucan V., Von Der Ohe J., Otte A., Hass R. (2016). Conditioned umbilical cord tissue provides a natural three-dimensional storage compartment as in vitro stem cell niche for human mesenchymal stroma/stem cells. *Stem Cell Research and Therapy*.

[35] Mandel K., Yang Y., Schambach A., Glage S., Otte A., Hass R. (2013). Mesenchymal stem cells directly interact with breast cancer cells and promote tumor cell growth in vitro and in vivo. *Stem Cells and Development*.

[36] Yang Y., Otte A., Hass R. (2015). Human mesenchymal stroma/stem cells exchange membrane proteins and alter functionality during interaction with different tumor cell lines. *Stem Cells and Development*.

[37] Otte A., Yang Y., Von Der Ohe J. (2016). SCCOHT tumors acquire chemoresistance and protection by interacting mesenchymal stroma/stem cells within the tumor microenvironment. *International Journal of Oncology*.

[38] Chung Y.-C., Wei W.-C., Hung C.-N. (2016). Rab11 collaborates E-cadherin to promote collective cell migration and indicates a poor prognosis in colorectal carcinoma. *European Journal of Clinical Investigation*.

[39] Friedl P., Wolf K. (2003). Tumour-cell invasion and migration: diversity and escape mechanisms. *Nature Reviews Cancer*.

[40] Friedl P., Wolf K. (2008). Tube travel: The role of proteases in individual and collective cancer cell invasion. *Cancer Research*.

[41] Achour O., Bridiau N., Kacem M. (2013). Cathepsin D activity and selectivity in the acidic conditions of a tumor microenvironment: utilization in the development of a novel Cathepsin D substrate for simultaneous cancer diagnosis and therapy. *Biochimie*.

[42] Bonnans C., Chou J., Werb Z. (2014). Remodelling the extracellular matrix in development and disease. *Nature Reviews Molecular Cell Biology*.

[43] Fonović M., Turk B. (2014). Cysteine cathepsins and extracellular matrix degradation. *Biochimica et Biophysica Acta*.

[44] Mohamed M. M., Sloane B. F. (2006). Cysteine cathepsins: multifunctional enzymes in cancer. *Nature Reviews Cancer*.

[45] Dennhöfer R., Kurschat P., Zigrino P. (2003). Invasion of melanoma cells into dermal connective tissue in vitro: Evidence for an important role of cysteine proteases. *International Journal of Cancer*.

[46] Skrzypczak M., Springwald A., Lattrich C. (2012). Expression of cysteine protease cathepsin L is increased in endometrial cancer and correlates with expression of growth regulatory genes. *Cancer Investigation*.

[47] Victor B. C., Anbalagan A., Mohamed M. M., Sloane B. F., Cavallo-Medved D. (2011). Inhibition of cathepsin B activity attenuates extracellular matrix degradation and inflammatory breast cancer invasion. *Breast Cancer Research*.

[48] Borgoño C. A., Diamandis E. P. (2004). The emerging roles of human tissue kallikreins in cancer. *Nature Reviews Cancer*.

[49] Sterbis J. R., Gao C., Furusato B. (2008). Higher expression of the androgen-regulated gene PSA/HK3 mRNA in prostate cancer tissues predicts biochemical recurrence-free survival. *Clinical Cancer Research*.

[50] Beaufort N., Debela M., Creutzburg S. (2006). Interplay of human tissue kallikrein 4 (hK4) with the plasminogen activation system: hK4 regulates the structure and functions of the urokinase-type plasminogen activator receptor (uPAR). *Biological Chemistry*.

[51] Deryugina E. I., Bourdon M. A., Reisfeld R. A., Strongin A. (1998). Remodeling of collagen matrix by human tumor cells requires activation and cell surface association of matrix metalloproteinase-2. *Cancer Research*.

[52] Hirashima K., Iyama K.-I., Baba Y. (2013). Differential expression of basement membrane type IV collagen *α*2 and *α*6 chains as a prognostic factor in patients with extrahepatic bile duct carcinoma. *Journal of Surgical Oncology*.

[53] Bertram C., Hass R. (2008). MMP-7 is involved in the aging of primary human mammary epithelial cells (HMEC). *Experimental Gerontology*.

[54] Bertram C., Hass R. (2009). Cellular senescence of human mammary epithelial cells (HMEC) is associated with an altered MMP-7/HB-EGF signaling and increased formation of elastin-like structures. *Mechanisms of Ageing and Development*.

[55] Chaturvedi S., Hass R. (2011). Extracellular signals in young and aging breast epithelial cells and possible connections to age-associated breast cancer development. *Mechanisms of Ageing and Development*.

[56] Itoh Y., Palmisano R., Anilkumar N., Nagase H., Miyawaki A., Seiki M. (2011). Dimerization of MT1-MMP during cellular invasion detected by fluorescence resonance energy transfer. *Biochemical Journal*.

[57] Lagoutte E., Villeneuve C., Lafanechère L. (2016). LIMK Regulates Tumor-Cell Invasion and Matrix Degradation Through Tyrosine Phosphorylation of MT1-MMP. *Scientific Reports*.

[58] Sabeh F., Ota I., Holmbeck K. (2004). Tumor cell traffic through the extracellular matrix is controlled by the membrane-anchored collagenase MT1-MMP. *Journal of Cell Biology*.

[59] Wolf K., Wu Y. I., Liu Y. (2007). Multi-step pericellular proteolysis controls the transition from individual to collective cancer cell invasion. *Nature Cell Biology*.

[60] Maquoi E., Assent D., Detilleux J., Pequeux C., Foidart J.-M., Noël A. (2012). MT1-MMP protects breast carcinoma cells against type i collagen-induced apoptosis. *Oncogene*.

[61] Sakamoto Y., Prudhomme S., Zaman M. H. (2011). Viscoelastic gel-strip model for the simulation of migrating cells. *Annals of Biomedical Engineering*.

[62] van Zijl F., Krupitza G., Mikulits W. (2011). Initial steps of metastasis: cell invasion and endothelial transmigration. *Mutation Research/Reviews in Mutation Research*.

[63] Enterline H. T., Coman D. R. (1950). The ameboid motility of human and animal neoplastic cells. *Cancer*.

[64] Friedl P., Zänker K. S., Bröcker E.-B. (1998). Cell migration strategies in 3-D extracellular matrix: Differences in morphology, cell matrix interactions, and integrin function. *Microscopy Research and Technique*.

[65] Friedl P., Gilmour D. (2009). Collective cell migration in morphogenesis, regeneration and cancer. *Nature Reviews Molecular Cell Biology*.

[66] Friedl P., Hegerfeldt Y., Tusch M. (2004). Collective cell migration in morphogenesis and cancer. *International Journal of Developmental Biology*.

[67] Kuriyama S., Yoshida M., Yano S. (2016). LPP inhibits collective cell migration during lung cancer dissemination. *Oncogene*.

[68] Lobastova L., Kraus D., Glassmann A. (2016). Collective cell migration of thyroid carcinoma cells: a beneficial ability to override unfavourable substrates. *Cellular Oncology*.

[69] Paňková K., Rösel D., Novotný M., Brábek J. (2010). The molecular mechanisms of transition between mesenchymal and amoeboid invasiveness in tumor cells. *Cellular and Molecular Life Sciences*.

[70] Wolf K., Mazo I., Leung H. (2003). Compensation mechanism in tumor cell migration: mesenchymal-amoeboid transition after blocking of pericellular proteolysis. *Journal of Cell Biology*.

[71] Giampieri S., Manning C., Hooper S., Jones L., Hill C. S., Sahai E. (2009). Localized and reversible TGF*β* signalling switches breast cancer cells from cohesive to single cell motility. *Nature Cell Biology*.

[72] Hernandez L., Magalhaes M. A. O., Coniglio S. J., Condeelis J. S., Segall J. E. (2011). Opposing roles of CXCR4 and CXCR7 in breast cancer metastasis. *Breast Cancer Research*.

[73] Günther K., Leier J., Henning G. (2005). Prediction of lymph node metastasis in colorectal carcinoma by expression of chemokine receptor CCR7. *International Journal of Cancer*.

[74] Mashino K., Sadanaga N., Yamaguchi H. (2002). Expression of chemokine receptor CCR7 is associated with lymph node metastasis of gastric carcinoma. *Cancer Research*.

[75] Müller A., Homey B., Soto H. (2001). Involvement of chemokine receptors in breast cancer metastasis. *Nature*.

[76] Lapteva N., Yang A.-G., Sanders D. E., Strube R. W., Chen S.-Y. (2005). CXCR4 knockdown by small interfering RNA abrogates breast tumor growth in vivo. *Cancer Gene Therapy*.

[77] Song N., Huang Y., Shi H. (2009). Overexpression of platelet-derived growth factor-BB increases tumor pericyte content via stromal-derived factor-1*α*/CXCR4 axis. *Cancer Research*.

[78] Karnoub A. E., Dash A. B., Vo A. P. (2007). Mesenchymal stem cells within tumour stroma promote breast cancer metastasis. *Nature*.

[79] Wagenblast E., Soto M., Gutiérrez-Ángel S. (2015). A model of breast cancer heterogeneity reveals vascular mimicry as a driver of metastasis. *Nature*.

[80] Alitalo K. (2011). The lymphatic vasculature in disease. *Nature Medicine*.

[81] Li S., Ma Y., Xie C. (2015). EphA6 promotes angiogenesis and prostate cancer metastasis and is associated with human prostate cancer progression. *Oncotarget*.

[82] Le Guelte A., Dwyer J., Gavard J. (2011). Jumping the barrier: VE-cadherin, VEGF and other angiogenic modifiers in cancer.. *Biology of the cell / under the auspices of the European Cell Biology Organization*.

[83] Aragon-Sanabria V., Pohler S. E., Eswar V. J., Bierowski M., Gomez E. W., Dong C. (2017). VE-Cadherin Disassembly and Cell Contractility in the Endothelium are Necessary for Barrier Disruption Induced by Tumor Cells. *Scientific Reports*.

[84] Weis S., Cui J., Barnes L., Cheresh D. (2004). Endothelial barrier disruption by VEGF-mediated Src activity potentiates tumor cell extravasation and metastasis. *The Journal of Cell Biology*.

[85] Minder P., Zajac E., Quigley J. P., Deryugina E. I. (2015). EGFR Regulates the Development and Microarchitecture of Intratumoral Angiogenic Vasculature Capable of Sustaining Cancer Cell Intravasation. *Neoplasia*.

[86] Joosse S. A., Pantel K. (2013). Biologic challenges in the detection of circulating tumor cells. *Cancer Research*.

[87] Camara O., Kavallaris A., Nöschel H., Rengsberger M., Jörke C., Pachmann K. (2006). Seeding of epithelial cells into circulation during surgery for breast cancer: the fate of malignant and benign mobilized cells. *World Journal of Surgical Oncology*.

[88] Pantel K., Speicher M. R. (2016). The biology of circulating tumor cells. *Oncogene*.

[89] Liu S., Ginestier C., Ou S. J. (2011). Breast cancer stem cells are regulated by mesenchymal stem cells through cytokine networks. *Cancer Research*.

[90] Mani S. A., Guo W., Liao M.-J. (2008). The epithelial-mesenchymal transition generates cells with properties of stem cells. *Cell*.

[91] Kreso A., Dick J. (2014). Evolution of the cancer stem cell model. *Cell Stem Cell*.

[92] Meacham C. E., Morrison S. J. (2013). Tumour heterogeneity and cancer cell plasticity. *Nature*.

[93] Visvader J. E., Lindeman G. J. (2008). Cancer stem cells in solid tumours: accumulating evidence and unresolved questions. *Nature Reviews Cancer*.

[94] Ye X., Tam W. L., Shibue T. (2015). Distinct EMT programs control normal mammary stem cells and tumour-initiating cells. *Nature*.

[95] Hanssen A., Wagner J., Gorges T. M. (2016). Characterization of different CTC subpopulations in non-small cell lung cancer. *Scientific Reports*.

[96] Mitchell M. J., King M. R. (2013). Computational and experimental models of cancer cell response to fluid shear stress. *Frontiers in Oncology*.

[97] Nagrath S., Sequist L. V., Maheswaran S. (2007). Isolation of rare circulating tumour cells in cancer patients by microchip technology. *Nature*.

[98] Alix-Panabières C., Pantel K. (2014). Challenges in circulating tumour cell research. *Nature Reviews Cancer*.

[99] Mu Z., Wang C., Ye Z. (2015). Prospective assessment of the prognostic value of circulating tumor cells and their clusters in patients with advanced-stage breast cancer. *Breast Cancer Research and Treatment*.

[100] Triantafillu U., Park S., Klaassen N., Raddatz A., Kim Y. (2017). Fluid shear stress induces cancer stem cell-like phenotype in MCF7 breast cancer cell line without inducing epithelial to mesenchymal transition. *International Journal of Oncology*.

[101] Maheswaran S., Haber D. A. (2010). Circulating tumor cells: a window into cancer biology and metastasis. *Current Opinion in Genetics and Development*.

[102] Steinert G., Schölch S., Niemietz T. (2014). Immune escape and survival mechanisms in circulating tumor cells of colorectal cancer. *Cancer Research*.

[103] Jurasz P., Alonso-Escolano D., Radomski M. W. (2004). Platelet–cancer interactions: mechanisms and pharmacology of tumour cell-induced platelet aggregation. *British Journal of Pharmacology*.

[104] Lian L., Li W., Li Z.-Y. (2013). Inhibition of MCF-7 breast cancer cell-induced platelet aggregation using a combination of antiplatelet drugs. *Oncology Letters*.

[105] Grignani G., Pacchiarini L., Ricetti M. M. (1989). Mechanisms of platelet activation by cultured human cancer cells and cells freshly isolated from tumor tissues. *Invasion and Metastasis*.

[106] Oleksowicz L., Mrowiec Z., Schwartz E., Khorshidi M., Dutcher J. P., Puszkin E. (1995). Characterization of tumor-induced platelet aggregation: the role of immunorelated GPIb and GPIIb IIIa expression by MCF-7 breast cancer cells. *Thrombosis Research*.

[107] Alonso-Escolano D., Strongin A. Y., Chung A. W., Deryugina E. I., Radomski M. W. (2004). Membrane type-1 matrix metalloproteinase stimulates tumour cell-induced platelet aggregation: Role of receptor glycoproteins. *British Journal of Pharmacology*.

[108] Cattaneo M. (2015). P2Y12 receptors: structure and function. *Journal of Thrombosis and Haemostasis*.

[109] Im J. H., Fu W., Wang H. (2004). Coagulation facilitates tumor cell spreading in the pulmonary vasculature during early metastatic colony formation. *Cancer Research*.

[110] Nieswandt B., Hafner M., Echtenacher B., Männel D. N. (1999). Lysis of tumor cells by natural killer cells in mice is impeded by platelets. *Cancer Research*.

[111] Placke T., Örgel M., Schaller M. (2012). Platelet-derived MHC class I confers a pseudonormal phenotype to cancer cells that subverts the antitumor reactivity of natural killer immune cells. *Cancer Research*.

[112] Padua D., Massagué J. (2009). Roles of TGFbeta in metastasis. *Cell Research*.

[113] Petrocca F., Visone R., Onelli M. R. (2008). E2F1-regulated microRNAs impair TGF*β*-dependent cell-cycle arrest and apoptosis in gastric cancer. *Cancer Cell*.

[114] Gorelik L., Flavell R. A. (2001). Immune-mediated eradication of tumors through the blockade of transforming growth factor-beta signaling in T cells. *Nature Medicine*.

[115] Yu M., Ting D. T., Stott S. L. (2012). RNA sequencing of pancreatic circulating tumour cells implicates WNT signalling in metastasis. *Nature*.

[116] Meng S., Tripathy D., Frenkel E. P. (2004). Circulating tumor cells in patients with breast cancer dormancy. *Clinical Cancer Research*.

[117] Yu M., Stott S., Toner M., Maheswaran S., Haber D. A. (2011). Circulating tumor cells: approaches to isolation and characterization. *The Journal of Cell Biology*.

[118] Reymond N., D'Água B. B., Ridley A. J. (2013). Crossing the endothelial barrier during metastasis. *Nature Reviews Cancer*.

[119] Gakhar G., Navarro V. N., Jurish M. (2013). Circulating tumor cells from prostate cancer patients interact with E-selectin under physiologic blood flow. *PLoS ONE*.

[120] Kienast Y., von Baumgarten L., Fuhrmann M. (2010). Real-time imaging reveals the single steps of brain metastasis formation. *Nature Medicine*.

[121] Zhu C., Yago T., Lou J., Zarnitsyna V. I., McEver R. P. (2008). Mechanisms for flow-enhanced cell adhesion.. *Annals of biomedical engineering*.

[122] Strilic B., Yang L., Albarrán-Juárez J. (2016). Tumour-cell-induced endothelial cell necroptosis via death receptor 6 promotes metastasis. *Nature*.

[123] Zen K., Liu D.-Q., Guo Y.-L. (2008). CD44v4 is a major E-selectin ligand that mediates breast cancer cell transendothelial migration. *PLoS ONE*.

[124] Reymond N., Im J. H., Garg R. (2012). Cdc42 promotes transendothelial migration of cancer cells through *β*1 integrin. *Journal of Cell Biology*.

[125] Leong H. S., Robertson A. E., Stoletov K. (2014). Invadopodia are required for cancer cell extravasation and are a therapeutic target for metastasis. *Cell Reports*.

[126] Felding-Habermann B., O'Toole T. E., Smith J. W. (2001). Integrin activation controls metastasis in human breast cancer. *Proceedings of the National Academy of Sciences of the United States of America*.

[127] Weber M. R., Zuka M., Lorger M. (2016). Activated tumor cell integrin *α*v*β*3 cooperates with platelets to promote extravasation and metastasis from the blood stream. *Thrombosis Research*.

[128] Hamilla S. M., Stroka K. M., Aranda-Espinoza H. (2014). VE-cadherin-independent cancer cell incorporation into the vascular endothelium precedes transmigration. *PLoS ONE*.

[129] Chambers A. F., Groom A. C., MacDonald I. C. (2002). Dissemination and growth of cancer cells in metastatic sites. *Nature Reviews Cancer*.

[130] Minn A. J., Kang Y., Serganova I. (2005). Distinct organ-specific metastatic potential of individual breast cancer cells and primary tumors. *Journal of Clinical Investigation*.

[131] Tang D. G. (2012). Understanding cancer stem cell heterogeneity and plasticity. *Cell Research*.

[132] Bhat-Nakshatri P., Appaiah H., Ballas C. (2010). SLUG/SNAI2 and Tumor Necrosis Factor Generate Breast Cells With CD44+/CD24- Phenotype. *BMC Cancer*.

[133] Keysar S. B., Jimeno A. (2010). More than markers: biological significance of cancer stem cell-defining molecules. *Molecular Cancer Therapeutics*.

[134] Kim H. J., Kim M.-J., Ahn S. H. (2011). Different prognostic significance of CD24 and CD44 expression in breast cancer according to hormone receptor status. *Breast*.

[135] Hosseini H., Obradovic M. M., Hoffmann M. (2016). Early dissemination seeds metastasis in breast cancer. *Nature*.

[136] Hass R., Gunji H., Datta R. (1992). Differentiation and retrodifferentiation of human myeloid leukemia cells is associated with reversible induction of cell cycle-regulatory genes. *Cancer Research*.

[137] Al-Hajj M., Wicha M. S., Benito-Hernandez A., Morrison S. J., Clarke M. F. (2003). Prospective identification of tumorigenic breast cancer cells. *Proceedings of the National Academy of Sciences of the United States of America*.

[138] Charafe-Jauffret E., Ginestier C., Iovino F. (2009). Breast cancer cell lines contain functional cancer stem sells with metastatic capacity and a distinct molecular signature. *Cancer Research*.

[139] Ginestier C., Hur M. H., Charafe-Jauffret E. (2007). ALDH1 is a marker of normal and malignant human mammary stem cells and a predictor of poor clinical outcome. *Cell Stem Cell*.

[140] Ginestier C., Liu S., Diebel M. E. (2010). CXCR1 blockade selectively targets human breast cancer stem cells in vitro and in xenografts. *Journal of Clinical Investigation*.

[141] Chen X., Li X., Zhao B. (2015). Dormancy activation mechanism of oral cavity cancer stem cells. *Tumor Biology*.

[142] Nishikawa S., Dewi D. L., Ishii H. (2012). Transcriptomic study of dormant gastrointestinal cancer stem cells. *International Journal of Oncology*.

[143] Celià-Terrassa T., Meca-Cortés Ó., Mateo F. (2012). Epithelial-mesenchymal transition can suppress major attributes of human epithelial tumor-initiating cells. *The Journal of Clinical Investigation*.

[144] Slade M. J., Payne R., Riethdorf S. (2009). Comparison of bone marrow, disseminated tumour cells and blood-circulating tumour cells in breast cancer patients after primary treatment. *British Journal of Cancer*.

[145] Gunasinghe N. P. A. D., Wells A., Thompson E. W., Hugo H. J. (2012). Mesenchymal-epithelial transition (MET) as a mechanism for metastatic colonisation in breast cancer. *Cancer and Metastasis Reviews*.

[146] Pattabiraman D. R., Bierie B., Kober K. I. (2016). Activation of PKA leads to mesenchymal-to-epithelial transition and loss of tumor-initiating ability. *Science*.

[147] Chao Y. L., Shepard C. R., Wells A. (2010). Breast carcinoma cells re-express E-cadherin during mesenchymal to epithelial reverting transition. *Molecular Cancer*.

[148] Bendoraite A., Knouf E. C., Garg K. S. (2010). Regulation of miR-200 family microRNAs and ZEB transcription factors in ovarian cancer: Evidence supporting a mesothelial-to-epithelial transition. *Gynecologic Oncology*.

[149] Brabletz S., Brabletz T. (2010). The ZEB/miR-200 feedback loop-a motor of cellular plasticity in development and cancer?. *EMBO Reports*.

[150] Hurteau G. J., Carlson J. A., Spivack S. D., Brock G. J. (2007). Overexpression of the microRNA hsa-miR-200c leads to reduced expression of transcription factor 8 and increased expression of E-cadherin. *Cancer Research*.

[151] Ghajar C. M., Peinado H., Mori H. (2013). The perivascular niche regulates breast tumour dormancy. *Nature Cell Biology*.

[152] Lawler J. (2002). Thrombospondin-1 as an endogenous inhibitor of angiogenesis and tumor growth. *Journal of Cellular and Molecular Medicine*.

